# Geographic trends in research output and citations in veterinary medicine: insight into global research capacity, species specialization, and interdisciplinary relationships

**DOI:** 10.1186/1746-6148-9-115

**Published:** 2013-06-12

**Authors:** Mary M Christopher, Ana Marusic

**Affiliations:** 1 School of Veterinary Medicine, 4206 VM3A, University of California–Davis, One Shields Ave, Davis, CA 95616, USA; 2 Department of Research in Biomedicine and Health, University of Split School of Medicine, Soltanska 2, 21000, Split, Croatia

**Keywords:** Agriculture, Bibliometrics, Economics, Education, Journal, Medicine, Research publication

## Abstract

**Background:**

Bibliographic data can be used to map the research quality and productivity of a discipline. We hypothesized that bibliographic data would identify geographic differences in research capacity, species specialization, and interdisciplinary relationships within the veterinary profession that corresponded with demographic and economic indices.

**Results:**

Using the SCImago portal, we retrieved veterinary journal, article, and citation data in the Scopus database by year (1996–2011), region, country, and publication in species-specific journals (food animal, small animal, equine, miscellaneous), as designated by Scopus. In 2011, Scopus indexed 165 journals in the veterinary subject area, an increase from 111 in 1996. As a percentage of veterinary research output between 1996 and 2010, Western Europe and North America (US and Canada) together accounted for 60.9% of articles and 73.0% of citations. The number of veterinary articles increased from 8815 in 1996 to 19,077 in 2010 (net increase 66.6%). During this time, publications increased by 21.0% in Asia, 17.2% in Western Europe, and 17.0% in Latin America, led by Brazil, China, India, and Turkey. The United States had the highest number of articles in species-specific journals. As a percentage of regional output, the proportion of articles in small animal and equine journals was highest in North America and the proportion of articles in food animal journals was highest in Africa. Based on principal component analysis, total articles were highly correlated with gross domestic product (based on World Bank data). The proportion of articles in small animal and equine journals was associated with gross national income, research and development, and % urban population, as opposed to the proportion of food animal articles, agricultural output, and % rural population. Co-citations linked veterinary medicine with medicine in the United States, with basic sciences in Eastern Europe and the Far East, and with agriculture in most other regions and countries.

**Conclusions:**

Bibliographic data reflect the demographic changes affecting veterinary medicine worldwide and provide insight into current and changing global research capacity, specialization, and interdisciplinary affiliations. A more detailed analysis of species-specific trends is warranted and could contribute to a better understanding of educational and workforce needs in veterinary medicine.

## Background

Veterinary medicine is a diverse profession with strong historic roots in war (horses) and agriculture (livestock for food and fiber) [1,2]. During the past few decades, the veterinary profession has undergone a profound shift in focus from agricultural animals to companion animals in the United States (US) and to a lesser extent in Western Europe [1-4]. Several demographic and socioeconomic factors have contributed to this shift, including consolidation of the livestock and food production industries [5,6], urbanization, changing social attitudes [4,7], a gender shift towards women in veterinary medicine [7,8], species-based tracking in veterinary curricula [9], and expanding clinical specialization [10]. The number of veterinary graduates entering food animal practice in the US has declined significantly since 1989 [11] and more than 75% of new graduates now enter small animal practice or specialized training in fields such as oncology and orthopedic surgery [12]. Today, 76.9% of veterinarians in the US work primarily with companion animals and 7.8% work primarily with food animals [13]. This shift has had major implications on educational and research programs; created shortages of veterinarians in laboratory animal medicine, research, food animal practice, regulatory veterinary medicine, and public health; and led to concerns about the profession’s ability to meet global animal health and food security needs [1-4,7]. Despite rapid globalization of the veterinary profession, our understanding of the scope and quality of these demographic changes in other regions of the world, their potential effects on veterinary education and research, and their relationship to quantitative demographic indicators is limited.

Veterinary journals reflect the research and clinical practice trends and priorities of the veterinary profession around the world. Bibliometric data for scientific publications can be used to assess journal quality, map worldwide trends in research productivity and quality, and evaluate interdisciplinary alliances [14,15]. Bibliometric analyses also can be used to mirror geographic trends within a discipline and provide insight into current and changing research priorities in different regions and countries. We hypothesized that bibliographic data would identify geographic differences and changes over time in veterinary research capacity, trends in species specialization, and interdisciplinary relationships in the veterinary profession that corresponded with demographic and economic indices. To assess this we evaluated veterinary journals indexed in the Scopus citation database. Scopus indexes a broader geographic range of journals and more core veterinary journals than other indexes and is the only database that designates journal subgroups based on species [16,17]. We compared overall and species-specific research productivity to demographic and economic variables for each country. We also assessed the frequency of co-citations between veterinary journals and journals in other disciplines. The results of this study could help identify the leading edge of global shifts in veterinary specialization and research and indicate possible underlying cultural contexts and evolving directions of the veterinary profession.

## Methods

Data from the Scopus database were retrieved between Dec 2011 and Sept 2012 by using the SCImago portal [18]. Countries were organized into eight geographic regions: Western Europe, Eastern Europe, Africa (Central and Southern Africa), North America (US and Canada), Latin America (including the Caribbean), Middle East (Middle East and North Africa), Asia, and the Pacific region. These geographic regions were the same as those designated in SCImago, with the exception of North Africa and the Middle East, which were grouped together (as grouped by the World Health Organization), because of the low number of articles from African regions and because North African countries are closely aligned with Middle Eastern countries with regard to agriculture and cultural attitudes about animals [19].

The most current journal data (2011) obtained for the subject area “Veterinary” included the SCImago Journal Rank (SJR) indicator, H-index, total and citable articles (last 3 years), total citations (last 3 years), self-citations (last 3 years), citations/article (last 2 years), number of cited and uncited articles, years of coverage, country of publication, publisher, and subject category (food animal [FA], small animal [SA], equine [EQ], and miscellaneous [MISC]). The SJR is a prestige metric of journal impact based on the Scopus database that considers both the quantity of citations (average number of citations received in a specific year by documents published in the journal in the previous three years) and the quality of citations (based on the scientific influence of the journals that cite them) [20]. The SJR has been shown to be a good alternative to the impact factor for journal evaluation, although journal ranks differ, in part because the SJR restricts the inflationary effect of self-citations [20,21]. The journal rankings used in this study were based on the SJR. The number and country of publication of veterinary journals indexed in 1999 (for 1996 citation data), 2003, and 2007 were recorded to document changes in the database over time. The 2011 impact factors for veterinary journals indexed in Scopus were obtained from Journal Citation Reports [22]. The Directory of Open Access Journals was queried to ascertain which of the journals were open-access [23] Language of publication was determined by examining journal websites and instructions for authors and through indexers.

Veterinary article data for regions and countries were obtained for the 14-year period 1996–2010 combined and for 1996 and 2010 (the most recent year for which data were available) separately. Data included the H index (for countries), total and citable articles, citations, citations/article, self-citations, number of cited and uncited articles, and % of articles with international collaboration. Percent international collaboration was defined as the proportion of articles whose author affiliations included more than one country. Regional data were also obtained for each individual year between 1996 and 2010. The number and percentage of articles in species-specific journals was recorded for each country and region from 1996 to 2010 and for the entire period combined. World data were calculated manually from regional data.

The following demographic and economic indicators were retrieved from the database of the World Bank [24]: gross domestic product (GDP; in current US$), gross national income (GNI; per capita, Atlas method, in current US$), agriculture value added (as a % of GDP), research and development (R&D; % of GDP), total population, % urban population, and % rural population. “Agriculture, value added” corresponds to the net output of agricultural sectors (forestry, hunting, fishing, crop cultivation, and livestock production). “Research and development” includes current and capital expenditures on basic and applied research and experimental development. Data were obtained for 2010 for all variables except R&D, for which 2009 data were used (too few countries had data reported for 2010). Where data were not available in 2010 (or 2009 for R&D), data from the most recent year available was used (but not earlier than 2000). The number of veterinary faculties in each country was obtained from the American Veterinary Medical Association [25] and the World Association of Veterinarians [26]. Where discrepancies occurred between sources, the highest number was used. The number of veterinary faculty per population and articles per population, GDP, and veterinary faculty were calculated.

Co-citation maps were visualized in SCImago for all countries for which data were available between 1996–97 and 2009–10. The discipline to which veterinary medicine was directly linked by co-citations in each year was recorded. In addition, co-citation links between veterinary subject categories (FA, SA, EQ, MISC) and other subject categories were also visualized and recorded. Where subject categories were linked to more than one discipline, both disciplines were recorded. The strength of co-citation links was not determined.

Data were collated and computations were done in Excel (Microsoft, Redmond, WA, USA). Data were analyzed statistically using JMP (v. 10.0.0, SAS Institute, Cary, NC, USA). Because most data were not normally distributed, median and range of values were reported. Principal component analysis was used to evaluate correlations among total articles, number and proportion of species-specific articles (using data from 1996–2010), and demographic and economic indicators. Statistical significance was set at P < 0.05.

## Results

### Veterinary journals in the Scopus database

The number of veterinary journals indexed by Scopus was 109 in 1996, 111in 1999, 116 in 2005, 128 in 2007, and 165 in 2011. The net increase (n = 56) in veterinary journals in 2011 as compared with 1996 came from Western Europe (n = 17), Asia (n = 14), Latin America (n = 9), Eastern Europe (n = 6), North America (n = 5), and the Middle East (n = 5). Veterinary journal citation data, impact factor, accessibility, language, and other data were tabulated for each region (Table 1). Citation data were incomplete for 14 journals (2 FA, 12 MISC). Of the 52,322 articles published in veterinary journals in the last three years, 46,297 (88.5%) were classified by Scopus as citable articles.

**Table 1:** **Veterinary journals indexed by Scopus in 2011**, by region of publication*

**Variable**	**Western Europe**	**Eastern Europe**	**Africa**	**North America**	**Latin America**	**Middle East**	**Asia**	**Pacific**	**World**
No. journals (% of world)	82 (49.7)	14 (8.4)	2 (1.2)	25 (15.2)	13 (7.9)	6 (3.6)	20 (12.1)	3 (1.8)	165 (100)
No. open-access (% of world)	6 (17.6)	4 (11.8)	2 (5.9)	1 (2.9)	11 (32.4)	4 (11.8)	6 (17.6)	0 (0)	34 (100)
Impact factor, median (range)†	1.26 (0.03-4.25)	0.49 (0.17-0.67)	0.51, 0.52	0.94 (0.29-3.32)	0.31 (0.18-0.61)	0.29 (0.19-0.48)	0.32 (0.02-1.21)	0.95 (0.05-1.16)	0.92 (0.02-4.25)
SJR, median (range)	0.05 (0.03-0.25)	0.03 (0.03-0.05)	0.04	0.05 (0.03-0.15)	0.03 (0.03-04)	0.03 (0.03)	0.03 (0.03-0.15)	0.05 (0.03-0.06)	0.04 (0.03-0.25)
Journal H-index, median (range)	19 (1-73)	9 (2-20)	20 (18, 22)	21 (1-62)	6 (2-16)	3 (1-14)	6 (1-22)	27 (10-31)	14 (1-73)
No. articles last 3 years (% of world)	27,906 (53.3)	3428 (6.6)	245 (0.5)	7375 (14.1)	4463 (8.5)	1112 (2.1)	7147 (13.7)	646 (1.2)	52,322 (100)
No. citations last 3 years (% of world)	20,077 (70.4)	680 (2.4)	64 (0.2)	4857 (17.0)	950 (3.3)	270 (0.9)	1379 (4.8)	236 (0.8)	28,513 (100)
Uncited articles last 3 years (% of articles)	17,723 (63.5)	2907 (84.8)	196 (80.0)	4970 (67.4)	3704 (83.0)	905 (81.4)	6170 (86.3)	504 (78.0)	37,079 (70.8)
Self-citations last 3 years, (% of citations)§	3739 (18.6)	146 (21.5)	12 (18.8)	912 (18.8)	226 (23.8)	119 (44.1)	141 (10.2)	35 (14.8)	5358 (18.8)
Citations/article last 2 years, median (range)	0.52 (0-1.93)	0.17 (0.08-0.43)	0.23 (0.17, 0.29)	0.42 (0-2.04)	0.08 (0-0.32)	0.13 (0.07-0.36)	0.11 (0-0.79)	0.51 (0-0.57)	0.28 (0-2.04)
Language of publication (No. journals)	English (55), German (7), German-English (5), French (4), French-English (2), English-German-French (1), Spanish (2), Spanish-English (1), Dutch (1), Dutch-English (1), Italian-English (2), Japanese-English (1)	English (11), Hungarian (1), Lithuanian-English (1), Polish-English (1)	English (1), English-Afrikaans (1)	English (25)	Portuguese-English (6), Portuguese-Spanish-English (3), Spanish-English (3), Spanish (1)	English (1), Turkish-English (3), Arabic-English (1), Hebrew-English (1)	English (19), Korean-English (1)	English (3)	English only (115), non-English only (16), mixed (34)
Species-specific journals	FA (11), SA (10), EQ (7)	FA (1), SA (1)	—	FA (3), SA (6)	—	—	FA (3), EQ (1)	SA (1)	FA (18), SA (18), EQ (8)

*Data for number of journals and species-specific journals, SJR and H-index, and articles (total documents) and citations are from Scopus, obtained via SCImago [18]. Regional location of journals was based on the publisher location indicated in Scopus in 2011. The number of open access journals was obtained from the Directory of Open Access Journals [23]. Impact factor data were obtained from Journal Citation Reports [22]. Language of publications was obtained from journal indexers, websites, and guidelines for authors.

†The number of journals in the Scopus database that had 2011 impact factors were as follows: Western Europe (65), Eastern Europe (6), Africa (2), North America (19), Latin America (4), Middle East (4), Asia (7), and Pacific (3).

§Refers to within-journal self-citations.

See Appendix for complete list of species-specific journals. *FA* Food Animal, *SA* Small Animals, *EQ* Equine. Other journals were categorized as Miscellaneous.

### Veterinary article output and citations by country and region

Veterinary article and citation data were reported for 193 countries in the 8 regions for the combined 14-year period of 1996–2010 (Table 2). All but 10 countries had ≥90% citable articles. As a percentage of veterinary research output, Western Europe and North America together accounted for 60.9% of total articles and 73.0% of citations; countries in these regions also had higher H indexes than those in other regions. Eastern Europe had the fewest citations per article, whereas the Pacific region (Australia and New Zealand) had the most. The Pacific region also had the lowest proportions of uncited articles and self-citations, whereas Asia (largely India) had the highest. Africa had the highest percentage of articles with international collaboration, whereas the Middle East had the lowest.

**Table 2:** **Veterinary article and citation data for 193 countries in eight geographic regions during 1996**-2010*

**Variable**	**Western Europe**	**Eastern Europe**	**Africa**	**North America**	**Latin America**	**Middle East**	**Asia**	**Pacific**	**World**
No. countries	25	22	46	2	42	20	26	10	193
Country H index, median (range)	34 (0-83)	8 (0-32)	5 (1-37)	83 (67, 98)	4 (0-41)	10 (1-67)	13 (1-47)	5 (0-59)	7 (0-98)
No. articles (% of world)	56,745 (34.8)	11,046 (6.8)	5229 (3.2)	42,568 (26.1)	13,173 (8.1)	10,567 (6.5)	23,922 (14.7)	6580 (4.0)	163,250 (100)
No. citations (% of world)	430,298 (40.0)	37,122 (3.4)	30,859 (2.8)	359,000 (33.0)	52,174 (4.8)	33,453 (3.0)	87,608 (8.0)	57,255 (5.3)	1,087,769 (100)
Uncited articles (% of articles in region)	13,847 (24.4)	3069 (27.8)	1179 (22.5)	9067 (21.3)	5016 (38.1)	4118 (39.0)	10,366 (43.3)	975 (14.8)	47,637 (29.2)
Self-citations (% of citations in region)†	36,270 (8.4)	2646 (7.1)	1500 (4.9)	28,650 (8.0)	3653 (7.0)	2331 (7.0)	10,625 (12.1)	2774 (4.8)	88,449 (8.1)
Citations/article, median (range)	8.69 (0.85-12.00)	3.86 (0.37-5.52)	7.26 (0.63-9.83)	9.72 (0.79-13.77)	7.26 (0.39-9.20)	4.33 (0.46-7.33)	5.01 (0.54-6.91)	9.90 (1.22-14.29)	7.26 (0.37-14.29)
Average % articles with international collaboration	31.2	20.4	52.9	23.0	32.2	18.7	24.3	39.0	30.2

*Data are from Scopus, obtained via SCImago [18]. Articles are total documents.

†Refers to within-country self-citations.

Regional trends were led by specific countries, and did not necessarily apply to individual trends for other countries in the region. In 2010, the US alone published 17.7% of all veterinary articles, followed by the United Kingdom (UK), India, Germany, Brazil, Canada, France, Turkey, and Japan (Table 3). Of countries with >100 articles during the 14-year period, the number of citations/article was highest for Iceland (12.9 citations/article), Denmark (12.6), Norway (11.6), Sweden (11.5), and Hong Kong (11.1). India and Iran had the highest proportion of uncited articles; Taiwan and Denmark had the least. India and Turkey had the highest proportion of self-citations.

**Table 3:** **Article and citation data** (**1996**-**2010**) **for top countries in each region**, based on H index*

**Region**	**Country and Rank**	**H Index**	**Articles**	**Citations**	**Citations per Article**	**% Self-Citations**	**% Uncited Articles**	**% Int’l Collaboration**	**% World (1996)**	**% World (2010)**	**% Change**
Western Europe	1. UK	83	12,888	134,410	9.2	30.1	27.8	34.3	8.5	7.4	–1.1
	2. France	65	6084	47,501	7.4	24.2	36.1	32.6	3.7	3.1	–0.6
	3. Netherland	63	4084	42,705	10.4	21.1	17.4	41.7	2.7	2.1	–0.6
	4. Germany	57	10,024	55,387	5.5	30.2	28.5	42.2	5.2	5.0	–0.2
	5. Denmark	57	2303	27,512	12.6	22.2	11.4	44.4	1.2	1.1	–0.1
Eastern Europe	1. Czech Rep	32	1636	9780	6.3	40.3	19.6	24.0	0.8	0.7	–0.1
	2. Hungary	31	1768	7236	4.0	26.9	38.1	36.6	1.4	0.8	–0.6
	3. Poland	30	4934	12,316	2.7	51.8	35.0	11.6	2.4	3.3	+0.9
	4. Slovakia	23	856	3657	4.2	31.4	28.4	32.0	0.6	0.3	–0.3
	5. Croatia	19	659	2071	3.7	28.2	35.7	28.3	0.4	0.4	0
Africa	1. So. Africa	37	1767	11,688	6.8	30.8	18.1	36.0	1.1	0.8	–0.3
	2. Kenya	27	584	4819	7.8	24.4	14.6	67.8	0.6	0.3	–0.3
	3. Ethiopia	23	476	2702	7.4	34.2	14.3	62.4	0.2	0.4	+0.2
	4. Tanzania	21	270	2118	8.0	23.6	15.4	72.0	0.2	0.1	–0.1
	5. Zimbabwe	21	267	2006	6.7	17.7	17.0	71.7	0.3	0.1	–0.2
North America	1. U.S.	98	35,208	316,515	9.0	47.0	21.2	22.8	24.4	17.7	–6.7
	2. Canada	67	6138	56,027	9.2	22.4	19.8	38.9	4.3	3.0	–1.3
Latin America	1. Brazil	41	8066	27,452	5.8	49.1	26.4	25.3	1.7	8.1	+6.4
	2. Argentina	34	1204	8216	7.9	22.0	20.5	44.0	0.6	0.8	+0.2
	3. Mexico	29	1442	7090	7.5	30.5	20.8	42.2	0.5	1.3	+0.8
	4. Uruguay	21	194	1659	9.4	22.3	17.3	60.2	0.1	0.2	+0.1
	5. Chile	20	688	2617	4.1	27.8	28.2	42.4	0.5	0.4	–0.1
Middle East	1. Israel	34	804	7287	9.3	18.3	14.8	32.3	0.5	0.5	0
	2. Turkey	24	5954	12,661	2.6	54.5	36.1	8.1	1.0	4.8	+3.8
	3. Iran	20	1663	3559	3.7	37.6	40.1	22.3	0.3	2.0	+1.7
	4. Egypt	23	663	3131	6.3	14.6	19.6	51.0	0.3	0.6	+0.3
	5. UAE	18	198	1284	6.6	17.8	15.5	55.4	0.2	0.1	–0.1
Asia	1. Japan	47	5113	35,082	7.2	32.2	16.5	24.0	3.1	2.3	–0.8
	2. China	34	2344	12,231	9.5	37.6	13.6	40.9	0.2	3.4	+3.2
	3. So. Korea	32	1719	7990	9.6	24.9	22.2	33.0	0.2	2.0	+1.8
	4. India	30	12,147	17,676	1.6	55.5	57.5	22.5	5.2	6.9	+1.7
	5. Taiwan	29	623	5493	10.9	23.7	11.0	22.6	0.2	0.6	+0.4
Pacific	1. Australia	59	4662	42,185	9.4	24.9	13.9	39.9	3.5	2.7	–0.7
	2. N Zealand	45	1794	17,037	9.6	28.5	13.4	43.7	1.1	0.9	–0.2

*Data are from Scopus, obtained via SCImago [18]. Articles are total documents. These countries together account for 84% of veterinary article output.

**Figure 1:**
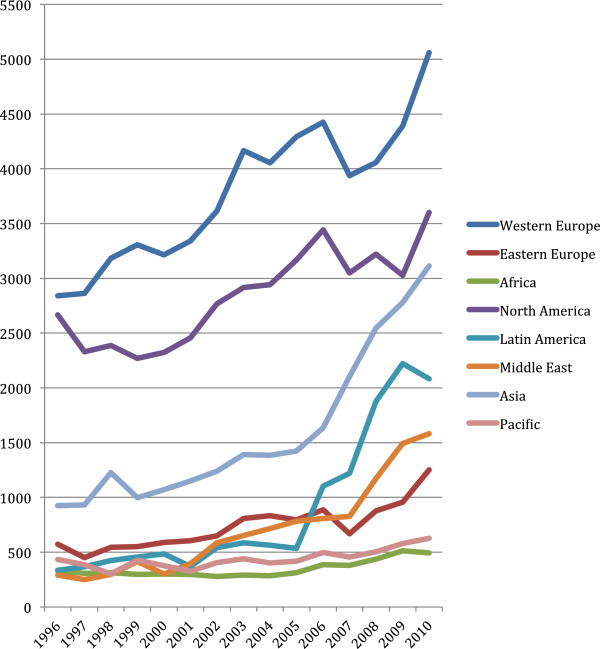
**Regional increases in the number of veterinary articles from 1996 to 2010.** Data were obtained from SCImago [18].

Worldwide, the number of veterinary articles published was 8815 in 1996 and 19,077 in 2010, for a net increase of 66.6% (Figure 1). The net increase in articles between 1996 and 2010 was highest in Asia (21.0% increase) followed by Western Europe (17.2%), Latin America (17.0%), the Middle East (12.3%), Eastern Europe (6.7%), North America (5.6%), Africa (1.5%), and the Pacific (1.4%). Between 1996 and 2010, China had the highest relative increase in article output (2767%, from 21 to 602 articles), followed by South Korea (1867%, from 18 to 354 articles), Iran (1207%, from 28 to 365 articles), Brazil (800%, from 162 to 1458 articles), Turkey (758%, from 100 to 858 articles), Italy (349%, 119 to 534 articles), Poland (157%, 233 to 599 articles), India (152%, 493 to 1241 articles), Germany (80%, 498 to 895 articles), and the US (36%, 2337 to 3179 articles). High relative increases also were seen in Mexico and Thailand. In Africa, Nigeria had the largest increase, from 49 to 117 articles. A net decrease in articles between 1996 and 2010 was found only in Zimbabwe (from 27 to 9 articles) and Kenya (from 60 to 45 articles). Changes in total article output from 1996 to 2010 coincided with concomitant changes in percentage worldwide article contribution in all countries except the US and Germany, which had increased numbers of articles, but decreased relative contributions to world output (Table 3).

### Species-specific output and relation to demographic and economic variables

**Figure 2:**
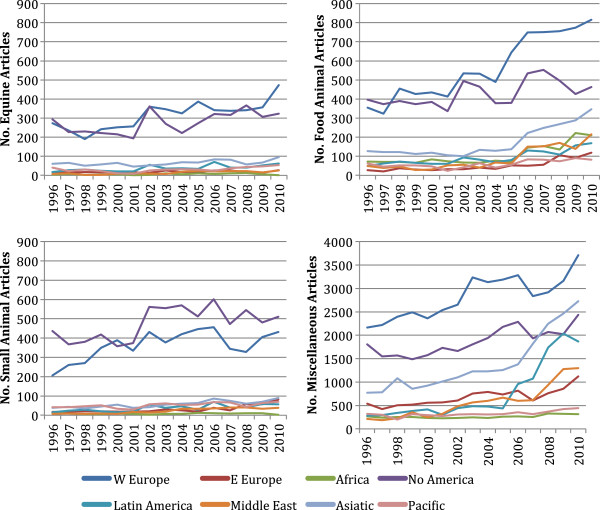
**Regional increases in the number of articles published in equine, food animal, small animal, and miscellaneous veterinary journals from 1996 to 2010.** Data were obtained from SCImago [18].

During the combined period of 1996–2010, 50,756 articles (31.1% of veterinary articles worldwide) were published in species-specific journals; of these, 15,587 (50.4%) were in FA journals, 15,715 (30.9%) were in SA journals, and 11,454 (22.5%) were in EQ journals (Table 4). The remaining 112,494 articles (68.9% of veterinary articles worldwide) were published in MISC journals. Western Europe and North America together published 64.0% of FA, 80.1% of SA, and 77.3% of EQ articles in species-specific journals in 1996–2010. Except for Asia, which contributed to 11% of world output in FA journals, other regions contributed <10% of articles in species-specific categories. The US had the most veterinary articles in all categories, with 5548 in FA journals (3.2-fold more than 2nd-ranked Germany; 23.5% of FA articles), 5957 in SA journals (4.8-fold more than 2nd-ranked UK; 37.9% of SA articles), 3506 in EQ journals (2.6-fold more than 2nd-ranked UK; 30.6% of EQ articles), and 22,957 in MISC journals (2.0-fold more than 2nd-ranked India; 20.4% of MISC articles)”. Articles in species-specific journals increased between 1996 and 2010 in all regions except Africa (Figure 2). The number of articles in 1996 and 2010 respectively was 1139 and 2421 in FA journals; 774 and 1280 in SA journals; 713 and 1063 in EQ journals; and 6361 and 13,390 in MISC journals. The UK had the largest net increase in SA articles (33 to 113), with high relative increases in Brazil, China, Poland, Taiwan, and Turkey. The UK also had the largest increase in EQ articles (84 to 124), with high relative increases in Brazil, China, and Taiwan. Germany had the largest increase in FA articles (59 to 193), with high relative increases in China, Ethiopia, Iran, and Turkey.

**Table 4:** **Regional publication output in veterinary species**-**specific journals (1996**-2010)*

**Region**	**Food Animal**			**Small Animal**			**Equine**		
	**No. articles**	**% of world**	**Top country**	**No. articles**	**% of world**	**Top country**	**No. articles**	**% of world**	**Top country**
Western Europe	8451	36.1	Germany	5451	34.7	UK	4716	41.2	UK
Eastern Europe	756	3.2	Poland	468	3.0	Poland	281	2.5	Poland
Africa	1492	6.4	Nigeria	97	0.6	So. Africa	72	0.6	So. Africa
North America	6645	27.6	US	7144	45.5	US	4144	36.2	US
Latin America	1383	5.9	Brazil	553	3.5	Brazil	570	5.0	Brazil
Middle East	1380	5.9	Iran	368	2.3	Turkey	227	2.0	Turkey
Asia	2581	11.1	India	853	5.4	Japan	982	8.6	Japan
Pacific	899	3.8	Australia	781	5.0	Australia	462	4.0	Australia
World	23,587	100	US	15,715	100	US	11,454	100	US

*Data are from Scopus, obtained via SCImago [18]. Top country is based on number of articles published.

**Figure 3:**
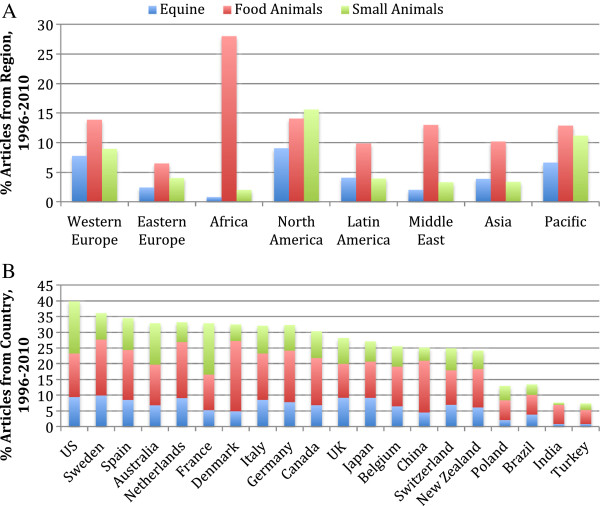
**Percentage of articles in species**-**specific veterinary journals within each region (A) and within the top 20 countries for species**-**specific article output (B).** Data were obtained from SCImago [18].

As a percentage of regional output, North America had the highest proportion of SA and EQ articles and Africa had the highest proportion of FA articles (Figure 3A). Within countries, the proportion of SA articles was highest in the US, France, and Australia (Figure 3B); the proportion of EQ articles was highest in Sweden; and the proportion of FA articles was highest in Denmark and the Netherlands. Less than 15% of articles from Turkey, India, Brazil and Poland were published in species-specific journals in the period 1996–2010.

**Figure 4:**
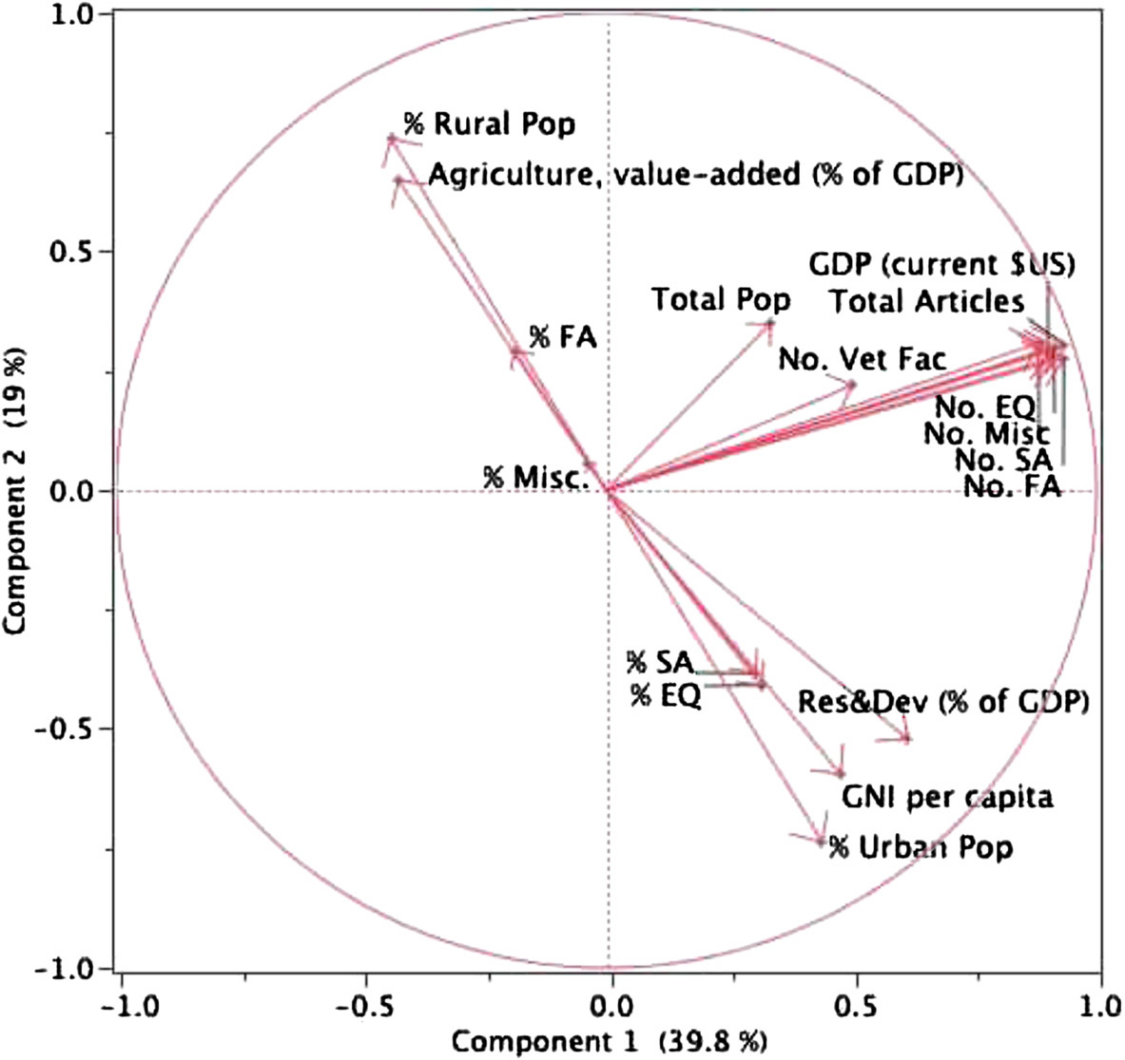
**Principal component analysis (correlation circle) of veterinary article output and demographic and economic variables.** The first principal component (horizontal axis) accounts for 39.8% of variation and comprises the highly correlated cluster of gross domestic product (GDP) and the number of articles (total, small animal [SA], equine [EQ], food animal [FA], miscellaneous [Misc]). The second principal component (vertical axis) accounts for 19% of variation and comprises the opposing clusters of agriculture value-added and % rural population (Pop.) versus gross national income (GNI) per capita, research and development (Res & Dev), and % urban Pop. Variables close to the center are less strongly correlated with variables far from the center in the same cluster because some information is carried on other axes. %SA, %EQ, %FA, and %Misc refer to the proportions of articles in species-specific journals. Bibliographic data [18] from 1996–2010 were used in the analysis; demographic and economic data were primarily from 2010 [24].

Demographic, economic, and veterinary faculty data were tabulated for the 193 countries that published veterinary articles from 1996–2010 (Table 5). Africa and Asia had the largest rural populations and the fewest articles per million people. When article output was normalized for GDP, the Pacific region ranked highest and the Middle East ranked lowest. Principal component analysis yielded three distinct clusters (Figure 4). Strong correlation (P<0.0001) was found between GDP, number of articles (total and species-specific), and number of veterinary faculties (component 1). A cluster consisting of agricultural output, % rural population, and % articles in FA journals was clearly and inversely separated from a cluster consisting of GNI per capita, % urban population, % R&D, and % articles in SA and EQ journals (component 2).

**Table 5:** **Demographic and economic data (2010) by region**, for countries that published veterinary articles from 1996 to 2010*

**Variable**	**Western Europe**	**Eastern Europe**	**Africa**	**North America**	**Latin America**	**Middle East**	**Asia**	**Pacific†**
No. countries	25	22	46	2	42	20	26	2
No. veterinary faculties	60	85	29	33	174	55	165	8
Total population (in millions)	413.5	337.3	842.6	343.5	588.4	454.0	3798.8	26.7
GDP, current US$ (in billions)	15.9	3.1	1.1	16.0	5.2	2.8	1.1	1.3
GNI per capita, current US$ (median)	42,970	7295	750	45,300	6115	6570	1870	37,775
Agriculture, value added, % of GDP (median)	1.8	6.4	23.9	1.5	6.3	7.3	16.2	4.0
R&D, % of GDP (median)§	1.8	0.7	0.2	2.4	0.4	0.6	0.2	1.8
% Rural population (median)	22.4	37.6	61.7	18.6	33.3	28.1	63.1	12.1
% Urban population (median)	77.6	62.5	38.4	81.5	66.7	71.9	36.9	88.0
Articles per million population (median)	133.4	7.3	3.4	160.0	8.9	9.7	2.4	329.7
Articles per million GDP (median)	3.4	1.7	3.3	3.4	1.2	1.0	1.1	8.9

*Data for veterinary faculties were obtained from the American Veterinary Medical Association [25] and the World Veterinary Association [26]. Data for population, gross domestic product (*GDP*), gross national income (*GNI*), agriculture value added, research and development (*R*&*D*), and % rural and urban populations were obtained from the World Bank [24]. Number of articles was for 1996-2010 from the Scopus database, obtained via SCImago [18].

†Data are for Australia and New Zealand.

§The number of countries for which R&D data were available: Western Europe (19), Eastern Europe (19), Africa (16), North America (2), Latin America (15), Middle East (8), Asia (13), Pacific (2).

**Figure 5:**
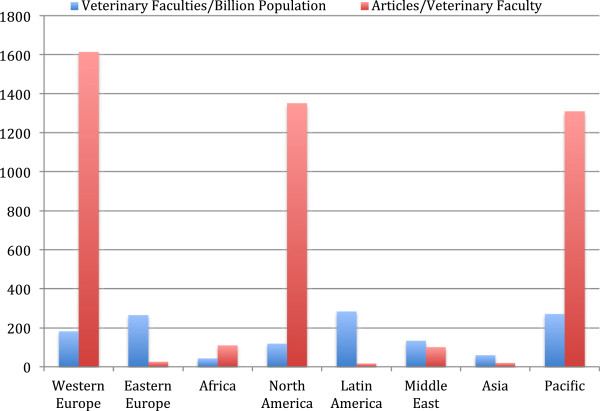
**Median number of veterinary faculties (normalized to population) and median article output (1996**–**2010) per faculty, by region.** Only countries with veterinary faculties were included in each region: Western Europe (n = 16), Eastern Europe (n = 21), Africa (n = 14), North America (n = 2), Latin America (n = 22), Middle East (n = 14), Asia (n = 21), and Pacific (n = 2).

The number of veterinary faculties was highest in Latin America, followed closely by Asia (Table 5). When normalized to population, Eastern Europe and Latin America had the most veterinary faculties, but publications per veterinary faculty were among the fewest (Figure 5). Between 1996 and 2010, the Netherlands, Denmark, and Sweden had the most articles per veterinary faculty (4457, 2443, and 2333, respectively), whereas, Russia, the Philippines, and Peru had the least articles per veterinary faculty (0.3, 5.7, and 13.4, respectively).

### Within-country co-citations between veterinary medicine and other disciplines

**Figure 6:**
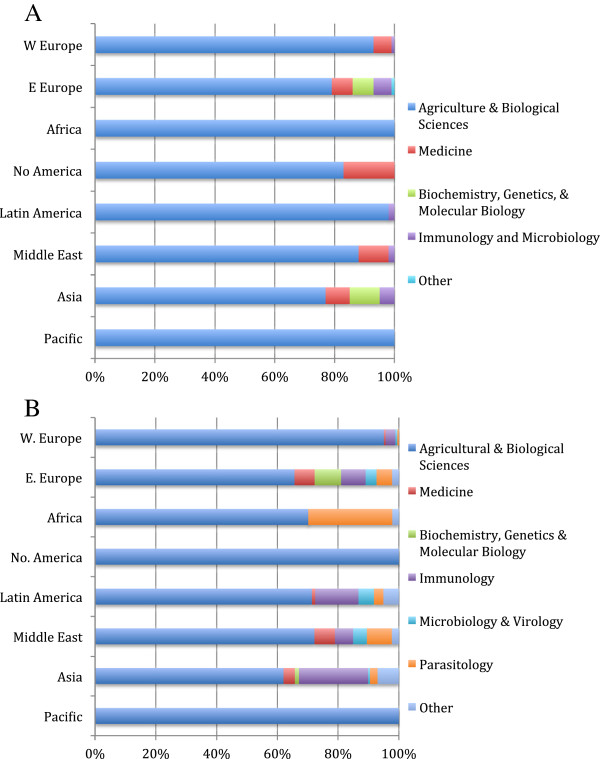
**Frequency of co-citation links between veterinary medicine and other disciplines, by region.** (**A**) Frequency of co-citation links between veterinary journals and journals in other subject areas. (**B**) Frequency of co-citation links between Miscellaneous veterinary journals and journals in other subject categories. Annual co-citation maps were available for up to 12 years (1998–2010) for 93 countries in Western Europe (n = 19), Eastern Europe (n = 20), Africa (n = 8), North America (n = 2), Latin America (n = 11), Middle East (n = 15), Asia (n = 16), and the Pacific (n = 2).

Co-citation maps were available for 93 countries for one or more years between 1998–99 and 2009–10. In all regions, veterinary articles were linked by co-citations primarily with Agriculture & Biological Sciences (ABS) journals (Figure 6A). Several Eastern European countries had co-citation links with Medicine and basic sciences; in the Middle East, Hong Kong and Singapore, veterinary articles were often linked with Medicine (Table 6). Co-citation links shifted from ABS to Medicine in Switzerland and the US between 2003 and 2005. Co-citations shifted from ABS to Biochemistry, Genetics, and Molecular Biology (BGM) in China, Taiwan, and South Korea between 2004 and 2006.

**Table 6:** **Changes in subject co**-citation links with veterinary journals over time in selected countries*

**Country**	**1998-99**	**1999-00**	**2000-01**	**2001-02**	**2002-03**	**2003-04**	**2004-05**	**2005-06**	**2006-07**	**2007-08**	**2008-09**	**2009-10**
United States	ABS	ABS	ABS	ABS	ABS	ABS	ABS	**MED**	**MED**	**MED**	ABS	**MED**
Switzerland	ABS	ABS	ABS	ABS	ABS	**MED**	**MED**	**MED**	**MED**	**MED**	ABS	**MED**
Austria	ABS	ABS	ABS	**MED**	ABS	ABS	**MED**	**MED**	ABS	ABS	ABS	ABS
Lithuania	ABS	ABS	ABS	**IMM**	**IMM**	ABS	ABS	ABS	ABS	ABS	ABS	ABS
Bulgaria	ABS	ABS	**IMM**	ABS	**IMM**	**MED**	**MED**	ABS	ABS	ABS	ABS	ABS
Serbia	–	–	–	–	–	–	–	–	**MED**	**BGM**	ABS	ABS
Russian Fed.	ABS	ABS	ABS	ABS	**BGM**	**MED**	**MED**	**BGM**	ABS	**BGM**	ABS	ABS
Romania	ABS	ABS	**BGM**	**BGM**	ABS	**BGM**	**BGM**	ABS	ABS	**MED**	ABS	ABS
Estonia	ABS	ABS	**BGM**	ABS	ABS	**BGM**	**BGM**	ABS	ABS	ABS	ABS	ABS
UAE	–	–	–	–	**MED**	**MED**	ABS	**MED**	**MED**	ABS	ABS	ABS
Kuwait	ABS	ABS	ABS	**MED**	**MED**	**MED**	**IMM**	ABS	ABS	**MED**	ABS	ABS
Algeria	–	–	ABS	ABS	ABS	**IMM**	ABS	**IMM**	ABS	ABS	ABS	ABS
China	ABS	ABS	ABS	ABS	ABS	ABS	ABS	ABS	**BGM**	**BGM**	**BGM**	**BGM**
South Korea	ABS	ABS	ABS	ABS	ABS	ABS	**BGM**	**BGM**	**BGM**	**BGM**	**BGM**	ABS
Taiwan	ABS	ABS	ABS	ABS	ABS	ABS	ABS	ABS	**BGM**	**BGM**	**BGM**	**BGM**
Hong Kong	**IMM**	ABS	**BGM**	**MED**	**MED**	**MED**	**MED**	**MED**	**MED**	**MED**	**MED**	**MED**
Singapore	ABS	**IMM**	**IMM**	**IMM**	ABS	ABS	**BGM**	**BGM**	ABS	**MED**	**MED**	**MED**
Thailand	**IMM**	ABS	ABS	ABS	**IMM**	ABS	ABS	ABS	ABS	ABS	ABS	ABS
Malaysia	**IMM**	**IMM**	ABS	ABS	ABS	ABS	ABS	ABS	ABS	ABS	ABS	ABS

*Subject area abbreviations are bolded for those years where co-citation links occurred between veterinary medicine and non-agricultural journal disciplines. Only countries with 50 or more veterinary articles between 1998 and 2010 and two or more years of co-citation links with non-agricultural journals are shown. *ABS* Agricultural & Biological Sciences, *MED* Medicine, *IMM* Immunology and Microbiology, *BGM* Biochemistry, Genetics, & Molecular Biology. Dashes indicate data were not available. Data were obtained from co-citation maps generated for each year and country via SCImago [18].

Within the Veterinary subject area, articles in FA journals were linked by co-citations almost exclusively with Animal Science & Zoology (ASZ) journals (within ABS); articles in EQ journals were linked with ASZ and other veterinary journals (data not shown). Articles in SA journals were linked with ASZ and Medicine, and, in the US, Western Europe (especially Switzerland), South Africa, and Australia, with MISC veterinary articles. In Asia, SA and EQ articles were linked with BGM. In Singapore, SA articles were linked with a variety of medical disciplines, including radiology & ultrasound, genetics, and biotechnology. Articles in MISC journals were linked by co-citations with Immunology, Parasitology, and Microbiology in all regions except North America and the Pacific (Figure 6B). In Japan, veterinary MISC articles were also linked with Marketing.

## Discussion

We used bibliographic data to provide a quantitative image of the current and evolving status of veterinary research capacity, species specialization, and interdisciplinary affiliations in regions and countries around the world between 1996 and 2011. Veterinary research productivity, closely associated with GDP, was strongly dominated by Western Europe and North America, but has increased rapidly in Asia, Latin America, and to a lesser extent in the Middle East and Eastern Europe. Western Europe and North America also led strongly in species specialization based on article output in FA, SA, and EQ journals. Reliance on the Scopus species-specific journal designations was a limitation, in that the majority of veterinary journals (in the MISC category) as well as other medical, animal science, and basic bioscience journals also publish species-specific veterinary articles. Because of this, the true magnitude and geographic distribution of species-specialized research remains uncertain; however, trends in this study were supported by principal component analysis, in which species emphasis aligned with opposing demographic and economic factors. Co-citation links supported strong alignment between veterinary medicine and agriculture in most countries, and also indicated distinct and evolving interdisciplinary links with medicine and basic sciences. Overall, these results provide a unique picture of the veterinary profession and its research capacity globally, and impel additional investigation into species-specific research output as an indicator of evolving education and workforce needs and priorities.

The Scopus database was a logical one to use for this study because it indexes the highest percentage of core veterinary journals [17], indexes geographically diverse journals [16], and to our knowledge is the only index to categorize veterinary journals according to species; data also are freely accessible through the SCImago portal, making it easy for readers to get detailed bibliographic data for their country, region, and discipline [18]. Compared with other indexes, Scopus is limited to more recent articles (beginning in 1995–96), however, this time period was relevant to our study and was balanced by the validity and large size of the Scopus co-citation journal database [21,27]. Although veterinary journals published in Western Europe, North America, and the Pacific included those with the highest impact factors, SJRs, and H indexes, the country of publication does not necessarily reflect the country of authors or affiliated societies, since many international journals are clustered within major publishers in Western Europe. Thus, although not all veterinary research is published in veterinary journals, Scopus provided a good cross-section of small to large journals of varying impact factors and national origin that reflected a wide range of veterinary research publications.

Western Europe and North America, led by the US and UK, accounted for more than 60% of veterinary articles and more than 70% of citations during the past 14 years, a regional dominance that has been described previously for other biomedical disciplines [14,15]. An earlier limited analysis of highly cited articles in veterinary journals in 2002 and 2003 found that 51% were from the US and UK [28]. In our study, notable growth in Italy and Spain has opened up a widening gap in research productivity between Western Europe and North America, and Asia is on a trajectory to exceed North America in veterinary publications in the near future. Indeed, Asia, with strong growth in India and China, surpassed North America in 2007 in the number of articles published in MISC veterinary journals. The increase in articles from Asia, Latin America, and the Middle East also likely reflected, in part, the addition of journals (and hence articles) from these regions to the Scopus database during the study period. Concomitant with growth in Asia, Latin America (Brazil), and the Middle East (Turkey, Iran), US contribution to world veterinary research output in veterinary medicine fell by nearly 7% from 1996 to 2010, supporting a trend noted in the 1990s and in other disciplines [29,30]. Although the decrease in the US and Germany was relative, decreases in other countries were absolute, and continued change is likely in the future.

Similar to bibliographic studies in other disciplines (which used different journal indexes and slightly different world regions), research output in the present study was strongly related to GDP [14,15,31]. The Pacific region had especially high article output relative to both GDP and population, as well as high quality articles, based on citation metrics. Notably, Africa produced about the same number of articles as Western Europe, North America, and the Pacific when article output was normalized to GDP. A possible explanation for this is the high average level of international collaboration (52%) for articles from Africa, suggesting that increasing international collaborations could be a fruitful way of enhancing veterinary research productivity in other regions, especially Eastern Europe and the Middle East. Uruguay and the UAE also had strong international collaboration, and ranked higher (based on H index) than other countries in their regions that produced more articles.

Despite increasing rates of publication, the quality and scientific impact of articles from Asia, Latin America, the Middle East, and Eastern Europe remain lower than in other regions, based on fewer citations, more self-citations and uncited articles, and lower citation metrics (impact factor, SJR, and H-index), parameters used as surrogates for research quality [32,33]. India, for example, published nearly as many articles as the UK during the study period, but had only about one-tenth of the citations. Language and accessibility of articles can affect citations, although a higher number of journals from these regions were open access. The H-index increases the longer articles are available to be cited, which favors well-established journals and countries with strong research infrastructures. Although citation-based metrics are commonly used to measure scientific impact, usage-based metrics (eg, online usage logs, social network analysis) may be just as important [34] and were not evaluated in the present study.

Another factor affecting regional differences in veterinary article output and quality appears to be the number of veterinary faculties relative to population, especially in Eastern Europe and Latin America, where limited resources are spread among a larger number of faculties than in other regions, making it more difficult to achieve critical mass and infrastructure for research. Although veterinary research is done not only in veterinary faculties, faculties are one indicator of a country’s capacity and structure for supporting veterinary research and education.

Countries with high total article output also tended to have high numbers of species-specific articles, and differences in urbanization and economic development correlated with species specialization within regions. The US had the highest overall rate of specialization (nearly 40% of US articles were in species-specific journals) and led the world in articles in SA-specific journals, followed by France and Western Europe in general. This SA (and to a lesser extent EQ) predominance was closely aligned with GNI and urbanization, consistent with expanding demand for companion animal specialty services in metropolitan areas, and the alignment of academic programs to meet research and training priorities for these species [2]. Notable differences in companion animal research output likely reflected differences in development between countries within a region. In Asia, for example, Japan ranked first in article output in SA and EQ journals, whereas India led in articles in FA journals. Similarly, South Africa ranked first in article output in SA and EQ journals, whereas Nigeria led in articles in FA journals. Increases in veterinary articles over time in most regions occurred in MISC and FA journals, but notable increases in SA and EQ journal articles were seen in Brazil, China, Poland, Taiwan, and Turkey. As previously noted, an important limitation of the Scopus classification of journals is that many species-specific articles are published in MISC journals (which comprised the largest group), as well as in journals in related subject areas. In addition, a few species-specific journals appeared to be misclassified (see Appendix). Thus, although the results of our study were consistent with what might be expected relative to demographic and economic indicators, additional studies are warranted to evaluate species-specific publications at the article rather than the journal level.

The strong co-citation affiliation between agriculture and veterinary journals, especially FA journals, was consistent with the importance of livestock production and disease in much of the world. Co-citations represent intellectual relationships between journals and have been used to study the specialty structure of science through the pattern of linkages, to monitor the development of scientific fields, and to assess developing interrelationships [27]. We noted several interesting patterns, including links between veterinary medicine and BGM in China, Taiwan, and South Korea (and some Eastern European countries), and links between veterinary medicine and medicine in several other countries. These patterns could be related to differences in culture, veterinary research infrastructure (relative to medicine and basic sciences), and geographic priorities, and warrant further investigation as potential indicators of shifts in species or interdisciplinary focus.

In the US and Switzerland, co-citation links provided tangible evidence of a recent shift in primary research affiliation from agriculture to medicine, although there could be more than one reason for this. The high output of articles in SA-specific journals, and parallels between SA and human medical practice (ie, residency/specialty training), make SA specialization a plausible explanation. If so, other countries in Western Europe with strong SA specialization, such as the UK and France, would be expected to undergo a similar shift in the near future. North America and Western Europe also had a high frequency of co-citation links between SA and MISC veterinary journals, the latter of which include veterinary specialty journals in subdisciplines like internal medicine, cardiology, dermatology, and ophthalmology. Increased laboratory animal, public health, and zoonotic disease research, such as that promoted by the One Health initiative [2,3], could also have contributed to co-citations with medicine in these countries. Again, a more detailed study with granularity at the article level is warranted to further assess the strength of co-citation affiliations and the evolving structure of veterinary research.

## Conclusions

In conclusion, bibliographic data documented geographic trends in research output and quality, species specialization, and interdisciplinary affiliations, and their relationship with demographic and economic factors that underlie many of the changes affecting the veterinary profession. Although conclusions about specialization based solely on species-specific journals are limited, it is clear that veterinary research output in the US and Western Europe has expanded notably in companion animal specialty areas, while research output in most regions of the world remains strongly focused on agriculture and food animal production. An emerging relevance gap along species lines could affect the ability of wealthy countries to contribute to veterinary training and research in developing countries, underlining concerns about the profession’s ability to meet global animal health and food security needs [1-4,7]. Facilitating international collaboration and focusing resources on fewer veterinary faculties also could help strengthen the quantity and quality of veterinary research and enable all regions to compete effectively. Future bibliographic studies that monitor and investigate evolving trends in veterinary and species-specific publications could improve our ability to understand and balance veterinary research and education needs globally.

## Appendix A

Journals (n = 165) designated as species-specific journals in the Scopus database, and the country of journal publication

### Food Animal (n = 18)

Animal Nutrition and Feed Technology (India)Annals of Animal Science (Poland)Applied Animal Behaviour Science (Netherlands)Avian Diseases (US)Avian Pathology (UK)Domestic Animal Endocrinology (Netherlands)International Journal of Dairy Science (Pakistan)International Journal of Poultry Science (Pakistan)International Journal of Probiotics and Prebiotics (US)Journal fur Verbraucherschutz und Lebensmittelsicherheit (Germany)Journal of Animal Physiology and Animal Nutrition (UK)Journal of Swine Health and Production (US)Preventive Veterinary Medicine (Netherlands)Small Ruminant Research (Netherlands)Theriogenology (Netherlands) (also listed in Small Animal and Equine)Tierarztliche Praxis Ausgabe G: Grosstiere-Nutztiere (Germany)Tropical Animal Health and Production (Netherlands)Veterinary Clinics of North America, Food Animal Practice (UK)

### Small Animal (n = 17)

Advances in Small Animal Medicine and Surgery (UK)Annals of Animal Science (Poland)Australian Veterinary Practitioner (Australia)Bulletin of the European Association of Fish Pathologists (UK)Journal of Avian Medicine and Surgery (US)Journal of the American Animal Hospital Association (US)Journal of Feline Medicine and Surgery (UK)Journal of Small Animal Practice (UK)PratiqueMedicaleetChirugicale de l’Animal de Compagnie (France)Pratique Vet (France), Kleintierpraxis (Germany)Theriogenology (Netherlands)Topics in Companion Animal Medicine (US)Veterinary Clinics of North America, Exotic Animal Practice (UK)Veterinary Clinics of North America, Small Animal Practice (UK)Veterinary Economics (US)Veterinary Medicine (US)Veterinary Technician (US)

### Equine (n = 8)

Equine Veterinary Education (UK)Equine Veterinary Journal (UK)Ippologia (Italy)Journal of Equine Science (Japan)Journal of Equine Veterinary Science (UK)Pferdeheilkunde (Germany)Theriogenology (Netherlands)Veterinary Clinics of North America, Equine Practice (UK)

### Miscellaneous (n = 125)

Acta Veterinaria (Serbia)Acta Veterinaria Brno (Czech Republic)Acta Veterinaria Hungarica (Hungary)Acta Veterinaria Scandinavica (Denmark)American Journal of Animal and Veterinary Sciences (US)American Journal of Veterinary Research (US)Animal Feed Science and Technology (Netherlands)Animal Science Journal (UK)Animal Science Papers and Reports (Poland)Animal Welfare (UK)Ankara Universitesi Veteriner Fakultesi Dergisi (Turkey)Annales de Medecine Veterinaire (Belgium)Archives of Animal Nutrition (UK)Archives of Veterinary Science (Brazil)Archivos de Medicina Veterinaria (Chile)Arquivo Brasileiro de Medicina Veterinaria e Zootecnia (Brazil)Asian Journal of Animal and Veterinary Advances (US)Australian Veterinary Journal (Australia)Berliner und Munchener Tierarztliche Wochenschrift (Germany)BMC Veterinary Research (UK)Brazilian Journal of Veterinary Research and Animal Science (Brazil)Buffalo Bulletin (Thailand)Bulgarian Journal of Agricultural Science (Bulgaria)Bulletin of the Veterinary Institute in Pulawy (Poland)CAB Reviews: Perspectives in Agriculture, Veterinary Science, Nutrition and Natural Resources (UK)California Cooperative Oceanic Fisheries, Investigations Reports (US)Canadian Journal of Veterinary Research (Canada)Canadian Veterinary Journal (Canada)Ciencia e Agrotecnologia (Brazil)Ciencia Rural (Brazil)Comparative Immunology, Microbiology and Infectious Diseases (Netherlands)Deutsche Tierarztliche Wochenschrift (Germany)Experimental Animals (Japan)Fish and Shellfish Immunology (US)In Practice (UK)Indian Journal of Animal Research (India)Indian Journal of Animal Sciences (India)Indian Veterinary Journal (India)International Journal of Applied Research in Veterinary Medicine (US)Iranian Journal of Veterinary Research (Iran)Iraqi Journal of Veterinary Sciences (Iraq)Irish Veterinary Journal (Ireland)Israel Journal of Veterinary Medicine (Israel)ITEA Informacion Tecnica Economica Agraria (Spain)Japanese Journal of Veterinary Research (Japan)Journal of Animal and Veterinary Advances (Pakistan)Journal of Applied Animal Research (India)Journal of Applied Animal Welfare Science (US)Journal of Aquatic Animal Health (US)Journal of Exotic Pet Medicine (Netherlands)Journal of Fish Diseases (UK)Journal of Medical Primatology (UK)Journal of the American Veterinary Medical Association (US)Journal of the South African Veterinary Association (South Africa)Journal of Veterinary Behavior: Clinical Applications and Research (Netherlands)Journal of Veterinary Cardiology (Netherlands)Journal of Veterinary Clinics (South Korea)Journal of Veterinary Dentistry (US)Journal of Veterinary Emergency and Critical Care (UK)Journal of Veterinary Internal Medicine (US)Journal of Veterinary Medical Education (US)Journal of Veterinary Medicine Series C: Anatomia Histologia Embryologia (UK)Journal of Veterinary Parasitology (India)Journal of Veterinary Science (Korea)Journal of Zhejiang University. Science. B. (China)Journal of Zoo and Wildlife Medicine (US)Kafkas Universitesi Veteriner Facultesi Dergisi (Turkey)Lab Animal (UK)Laboratory Animals (UK)Large Animal Review (Italy)Livestock Science (Netherlands)Magyar Allatorvosok Lapja (Hungary)Medical and Veterinary Entomology (UK)Medical Mycology (UK)Medicina Veterinaria (Spain)Medycyna Weterynaryjna (Poland)New Zealand Veterinary Journal (New Zealand)Onderstepoort Journal of Veterinary Research (South Africa)Pakistan Veterinary Journal (Pakistan)Pesquisa Veterinaria Brasileira (Brazil)Philippine Journal of Veterinary Medicine (Philippines)Point Veterinaire (France)Polish Journal of Veterinary Sciences (Poland)Praktische Tierarzt (Germany)Research in Veterinary Science (UK)Revista Brasileira de Parasitologia Veterinaria (Brazil)Revista Cientifica de la Facultad de Ciencias Veterinarias de la Universidad del Zulia (Venezuela)Revista Colombiana de Ciencias Pecuarias (Colombia)Revista MVZ Cordoba (Spain)RevistaVeterinaria (Argentina)Revue de Medecine Veterinaire (France)Scandinavian Journal of Laboratory Animal Science (Denmark)Schweizer Archiv fur Tierheilkunde (Switzerland)Slovenian Veterinary Research (Slovenia)Tecnica Pecuaria en Mexico (Mexico)Thai Journal of Veterinary Medicine (Thailand)Tierarztliche Praxis Ausgabe K: Kleintiere – Heimtiere (Germany)Tierarztliche Praxis. Supplement (Germany)Tierarztliche Umschau (Germany)Tijdschrift voor Diergeneeskunde (Netherlands)Transboundary and Emerging Diseases (Germany)Turkish Journal of Veterinary and Animal Sciences (Turkey)Veterinaria Mexico (Mexico)Veterinarija ir Zootechnika (Lithuania)Veterinarni Medicina (Czech Republic)Veterinarski Arhiv (Croatia)Veterinary Anaesthesia and Analgesia (UK)Veterinary and Comparative Oncology (UK)Veterinary and Comparative Orthopaedics and Traumatology (Germany)Veterinary Clinical Pathology (US)Veterinary Dermatology (UK)Veterinary Immunology and Immunopathology (Netherlands)Veterinary Journal (UK)Veterinary Microbiology (Netherlands)Veterinary Parasitology (Netherlands)Veterinary Pathology (UK)Veterinary Practitioner (India)Veterinary Radiology and Ultrasound (UK)Veterinary Record (UK)Veterinary Research (France)Veterinary Research Communications (Netherlands)Veterinary Surgery (UK)VlaamsDiergeneeskundigTijdschrift (Belgium)Wiener Tierarztliche Monatsschrift (Austria)

## Abbreviations

ABS: Agricultural and Biological Sciences; ASZ: Animal Science and Zoology; BGM: Biochemistry, Genetics, and Molecular Biology; EQ: Equine; FA: Food animal; GDP: Gross domestic product; GNI: Gross national income; MISC: Miscellaneous; R&D: Research and development; SA: Small animal; UK: United Kingdom; US: United States.

## Competing interests

The authors declare that they have no competing interests.

## Authors’ contributions

MMC defined the scope and hypothesis of the study, contributed to study design and data interpretation, acquired and analyzed the data, and drafted the manuscript. AM inspired the study, contributed to study design and data interpretation, and critically revised the manuscript. All authors read and approved the final manuscript.
